# Mammalian oocytes are targets for prostaglandin E2 (PGE2) action

**DOI:** 10.1186/1477-7827-8-131

**Published:** 2010-11-01

**Authors:** Diane M Duffy, Lynda K McGinnis, Catherine A VandeVoort, Lane K Christenson

**Affiliations:** 1 Department of Physiological Sciences, Eastern Virginia Medical School, Norfolk, VA 23507, USA; 2 Department of Molecular and Integrative Physiology, University of Kansas Medical Center, Kansas City, KS 66160, USA; 3 California National Primate Research Center, University of California, Davis, CA, 95616, USA; 4 Department of Obstetrics and Gynecology, School of Medicine, University of California, Davis, CA, 95616, USA

## Abstract

**Background:**

The ovulatory gonadotropin surge increases synthesis of prostaglandin E2 (PGE2) by the periovulatory follicle. PGE2 actions on granulosa cells are essential for successful ovulation. The aim of the present study is to determine if PGE2 also acts directly at the oocyte to regulate periovulatory events.

**Methods:**

Oocytes were obtained from monkeys and mice after ovarian follicular stimulation and assessed for PGE2 receptor mRNA and proteins. Oocytes were cultured with vehicle or PGE2 and assessed for cAMP generation, resumption of meiosis, and in vitro fertilization.

**Results:**

Germinal vesicle intact (GV) oocytes from both monkeys and mice expressed mRNA for the PGE2 receptors EP2, EP3, and EP4. EP2 and EP4 proteins were detected by confocal microscopy in oocytes of both species. Monkey and mouse oocytes responded to PGE2 as well as agonists selective for EP2 and EP4 receptors with elevated cAMP, consistent with previous identification of EP2 and EP4 as Gαs/adenylyl cyclase coupled receptors. Incubation of mouse GV stage oocytes with PGE2 delayed oocyte nuclear maturation in vitro, but PGE2 treatment did not alter the percentage of mouse oocytes that fertilized successfully. PGE2 treatment also decreased the percentage of monkey oocytes that resumed meiosis in vitro. In contrast with mouse oocytes, the percentage of monkey oocytes which fertilized in vitro was lower after treatment with PGE2. Monkey oocytes with intact cumulus showed delayed nuclear maturation, but fertilization rate was not affected by PGE2 treatment.

**Conclusions:**

Monkey and mouse oocytes express functional PGE2 receptors. PGE2 acts directly at mammalian oocytes to delay nuclear maturation. Surrounding cumulus cells modulate the effect of PGE2 to alter subsequent fertilization.

## Background

The ovulatory surge of luteinizing hormone (LH) stimulates events within the dominant ovarian follicle which lead to ovulation. One such action of LH is to increase PGE2 levels within the follicle [1]. PGE2 stimulates ovulatory events such as expansion of cumulus granulosa cells and enhanced expression of proteases associated with follicle rupture [2]. Blockade of PGE2 production within the follicle [3,4] or genetic manipulation which disrupts PGE2 production or PGE2 receptor expression [5,6] can prevent ovulation, further highlighting the essential role of receptor-mediated actions of PGE2 in mammalian ovulation.

PGE2 acts via G-protein coupled EP receptors [7]. Four distinct EP receptors (EP1, EP2, EP3, and EP4) have been identified. To date, examination of follicular EP receptor expression has focused on granulosa cells, with both cumulus and mural granulosa cells shown to express multiple types of EP receptors [8,9]. While PGE2 acts at both cumulus and mural granulosa cells of ovulatory follicles to promote periovulatory events [10,11], comparatively little is known about the oocyte as a target for PGE2 action. Detection of EP2 mRNA in bovine oocytes has been reported [12]. In contrast, a recent report suggests that mouse oocytes do not express EP receptor proteins [10]. The purpose of this study is to determine if the mammalian oocytes express EP receptors capable of signal transduction and modulation of oocyte function.

## Methods

### Monkey studies

#### Oocytes

Oocytes were obtained from adult female cynomolgus macaques (Macaca fascicularis) at Eastern Virginia Medical School (EVMS). All animal protocols were approved by the EVMS Animal Care and Use Committee and were conducted in accordance with the NIH Guide for the Care and Use of Laboratory Animals. Adult females with regular menstrual cycles were maintained as previously described and checked daily for menstruation; the first day of menstruation was designated day 1 of the menstrual cycle [13]. Blood samples were obtained under ketamine chemical restraint (10 mg/kg body weight) by femoral or saphenous venipuncture, and serum was stored at -20°C.

A controlled ovarian stimulation model developed for the collection of multiple oocytes for in vitro fertilization was used as previously described [13]. Recombinant human FSH (r-hFSH, 90 IU/day, Organon Pharmaceuticals, a part of Schering-Plough, now Merck & Co., Whitehouse Station, NJ) was administered for 6-8 days, followed by administration of r-hFSH plus r-hLH (Serono Reproductive Biology Institute, Rockland, MA, 60 IU/day) for 2-3 days to stimulate the growth of multiple preovulatory follicles. A GnRH antagonist (Ganirelix (30 μg/kg body weight; Merck) or Antide (0.5 mg/kg body weight; Serono)) was also administered daily to prevent an endogenous ovulatory LH surge. Adequate follicular development was monitored by serum estradiol levels and ultrasonography [14]. At aseptic surgery, each follicle was pierced with a 22-gauge needle, and the aspirated contents of all follicles larger than 4 mm in diameter were pooled.

In the laboratory, aspirates were subjected to centrifugation to pellet the oocytes and granulosa cells. Cells were resuspended in TALP-HEPES media and maintained at 37°C while oocytes were mechanically removed [14]. For assessment of EP receptor mRNAs, oocytes were treated with 1.0% hyaluronidase (weight/volume; w/v) for 15 seconds [15], followed by 0.5% pronase (w/v) for 2 minutes, and then rinsed by several transfers through clean medium to ensure granulosa cell removal. For cAMP analysis and culture of oocyte without cumulus, oocytes were treated with hyaluronidase only; remaining granulosa cells were removed with Stripper tips (MidAtlantic Diagnostics, Mount Laurel, NJ), and removal of granulosa cells was confirmed visually using a light microscope. For immunofluorescent detection of EP receptor proteins and culture of oocytes with intact cumulus, cumulus-oocyte complexes were handled gently to ensure that cumulus granulosa cells were not removed. All oocytes examined in this study were collected at the germinal vesicle (GV) stage of nuclear maturation.

#### Sperm

Adult male cynomolgus monkeys (Macaca fascicularis) were housed at the California National Primate Research Center (CNPRC) as described previously for rhesus monkeys [16]. All procedures for maintenance and handling of the animals were approved by the Institutional Animal Use and Care Administrative Advisory Committee at the University of California at Davis. Experiments were conducted in accordance with the NIH Guide for Care and Use of Laboratory Animals. Ejaculated semen samples were collected from 2 adult males that were individually caged at the CNPRC with lights on from 0600 to 1800 hours at 25°C to 27°C. The males were trained to chair restraint, and semen was collected by direct penile stimulation with a Grass 6 stimulator (Grass Medical Instruments, Quincy, Massachusetts) equipped with electrocardiogram pad electrodes (30-50 V, 20 ms duration, 18 pulses s-1) [17]. Samples were allowed to liquefy for 30 minutes before processing.

The sperm cryopreservation method used was identical to that previously published for rhesus monkey sperm [18]. In brief, a Styrofoam box (inside dimensions: 33 × 24 × 23 cm) was filled with a depth of 4 cm liquid nitrogen and a 1-cm-thick or 0.4-cm-thick Styrofoam "boat" was floated on top of it for 10 minutes. Straws were then placed on top of the "boat" for 10 minutes before being plunged into liquid nitrogen. The extender used was TEST-20% yolk-3% glycerol (w/v). The average cooling rate from -10°C to -70°C was about 220°C min-1 for the 1-cm boat.

#### Reverse transcription-PCR (RT-PCR)

Total RNA was obtained from 1-3 oocytes/monkey using Trizol reagent (Invitrogen, Rockville, MD) and concentrated to 1 μl final volume with vacuum concentration. One μl of 1 μM T7 (dT)_24 _promoter primer (5'GGCCAGTGAATTGTAATACGACTCACTATAGGGAGGCGGTTTTTTTTTTTTTTTTTTTTTTTT 3') was annealed at 70°C for 6 minutes. First strand and second strand cDNA synthesis was performed using the SuperScript II Choice Reverse Transcription system (Invitrogen). Following ammonium acetate precipitation, the cDNA was resuspended in 4 μl nuclease free water and in vitro-transcribed using the MegaScript T7 kit (Ambion) for 5 hours. The amplified cDNA was purified using the RNA clean-up procedure from the RNeasy Mini purification kit (Qiagen). Purified cDNA was vacuum concentrated and reverse transcribed as described previously [19].

Multiple mRNAs were analyzed in each monkey oocyte preparation by RT-PCR using a Roche LightCycler (Indianapolis, IN). PCR was performed using the FastStart DNA Master SYBR Green I kit (Roche). Primers for amplification of EP receptor cDNAs were designed based on cynomolgus monkey sequences using LightCycler Probe Design software (Roche); PCR primers, reaction conditions, and monkey sequence accession numbers were previously published [8]. β-actin mRNA was also detected by PCR to ensure successful amplification of each oocyte preparation. To ensure that oocytes were free of granulosa cells, only preparations negative for aromatase mRNA (expressed by cumulus granulosa cells but not oocytes [20]) were used for assessment of EP mRNAs. Intra- and inter-assay coefficients of variation for RT-PCR were less than 10%.

#### Immunofluorescent detection of EP receptors

Oocytes with attached cumulus cells were fixed in 4% paraformaldehyde (w/v) in phosphate buffered saline, pH 7.4 (PBS) for 20 min at room temperature. After washing in PBS, oocytes were blocked by incubation in PBS containing 0.1% Triton-X100 (volume/volume; v/v) and 5% goat serum (v/v; Vector Laboratories, Burlingame, CA) for 1 hour at room temperature then overnight at 4C. Primary antibody directed against a single EP receptor (2.0 μg/ml, rabbit polyclonal, Cayman Chemical, Ann Arbor, MI) was prepared in PBS+0.1% Triton-X100; oocytes were incubated in primary antibody solution for 2 hours at room temperature, washed in PBS, then incubated in Alexa-Fluor 488-conjugated secondary antibody directed against rabbit IgG (1:1000 dilution in PBS+0.1% Triton-X100, Molecular Probes, Eugene, OR) for 1 hour at room temperature before washing in PBS and mounting in aqueous mounting medium (Vectashield, Vector). Either propidium iodide or DAPI was included in the mounting medium to stain DNA and aid in locating oocytes for microscopy. In each experiment, the primary antibody was omitted for at least one oocyte such that these oocytes were incubated with PBS+0.1% Triton X-100 only. In other experiments, the primary antibody was preabsorbed with the peptide used to generate the antibody (2 μg/ml antibody + 6-10 μg/ml peptide; Cayman) for 2 hours at room temperature before incubation with oocytes.

Monkey oocytes were imaged using a Zeiss 510 scanning laser confocal microscope with LSM5 software for image acquisition (Carl Zeiss, Inc. Thornwood, NY). Fluorescent imaging was performed using 488 nm excitation with a 505/550 band-pass filter to image EP receptor fluorescence with gain of 595. Each oocyte was imaged as a Z-stack of approximately 20 images, each 0.5 μm in thickness. Two oocytes from each of a minimum of 3 animals were imaged for each primary antibody; 1 oocyte incubated with no primary antibody was imaged for each animal. For each primary antibody used, preabsorption was performed with oocytes possessing attached cumulus cells from at least 1 animal. Successful detection of all EP proteins in monkey kidney with these primary antibodies as well as elimination of immunofluorescence with preabsorption was previously reported [8].

#### Cyclic AMP generation

Monkey oocytes (6-10/group) were placed in 50 μl drops of TALP medium [14] containing either PGE2 (10^-6^M, Cayman), the EP2 selective agonist butaprost (10^-5^M, Cayman), the EP4 selective agonist PGE1 alcohol (10^-7^M, Cayman), or vehicle (0.06% DMSO; v/v). EP receptor agonist concentrations were selected to achieve maximal receptor occupancy while minimizing interaction with nontargeted EP receptors [7,8]. Oocytes without cumulus were cultured under oil in a humidified cell culture incubator (37°C, 5% O2, 95% CO2) for 3 hours. Media + oocytes was stored at -20°C until assayed for cAMP by EIA (Cayman) using the acetylation protocol according to manufacturer's instructions. Intra- and inter-assay coefficients of variation were 11.6% and 11.2%, respectively. Data are expressed as fmol cAMP/oocyte.

No granulosa cells were seen attached to oocytes prior to initiation of cAMP generation experiments. Preliminary experiments confirm that cAMP generation by contaminating granulosa cells cannot account for cAMP measured in oocyte cultures. Granulosa cells cultured in the absence of EP agonists produced approximately 1 × 10^-19 ^mol cAMP/cell·hour, while cells cultured in the presence of PGE2 produced approximately 3 × 10^-19 ^mol cAMP/cell·hour, indicating that contamination with about 10,000 cells/50 μl drop of TALP medium would be needed to produce cAMP as measured in oocyte cultures.

#### In vitro maturation and fertilization of monkey oocytes

Oocytes were cultured in media containing PGE2 or vehicle (DMSO) as described for cAMP generation. After 30 hours in vitro, the stage of nuclear maturation (germinal vesicle (GV), germinal vesicle break down (GVBD), or meiosis II (MII) was recorded. Each oocyte was moved to a fresh drop of TALP medium (without PGE2 or vehicle) for in vitro fertilization. Semen from cynomolgus macaques was thawed, washed, and activated as previously described [21,22]. Hypermotile sperm (10,000 per oocyte) were added to each drop of media. The next morning (about 20 hours later), oocytes were moved to fresh drops of media without PGE2 or vehicle and observed each day for the next 2 days. Fertilization was assessed by observation of a second polar body. Cleavage to form an embryo of 2 or more cells was also noted. Embryos were stained with DAPI to confirm that each cell contained DNA (not shown).

### Mouse studies

#### Animals

CF1 female mice aged 19-21 days old and B6D2F1 males aged 3-6 months were purchased from Harlan (Indianapolis, IN USA) and housed in a temperature and light-controlled room on a 14 hour light: 10 hour dark cycle. Experiments were approved by the University of Kansas Medical Center Animal Care and Use Committee and conducted in accordance with the NIH Guide for the Care and Use of Laboratory Animals.

#### In vitro maturation and fertilization of mouse oocytes

Female mice were stimulated with 5 IU equine chorionic gonadotropin (eCG; Calbiochem, San Diego CA USA). Ovaries were collected at 42-46 hours post-eCG at sacrifice by isofluorothane inhalation anesthesia followed by cervical dislocation. Cumulus-enclosed oocytes were released from large antral follicles into HEPES-buffered KSOM (FHM, Chemicon-Millipore, Billerica MA) and 4 mg/ml BSA (mFHM). Cumulus cells were manually stripped by repeated pipetting with a pulled glass pipette.

In vitro maturation was performed as previously described in K-MAT medium (KSOM^AA ^medium supplemented on the day of cultures with 1 mM glycyl-glutamine, 0.23 mM pyruvate, 4 mg/ml BSA, 0.6 mM L-cysteine, 0.5 mg/ml D-glucosamine, 0.02 μM ascorbate, 0.1% insulin-transferrin-selenium (ITS; Sigma Corp, St Louis MO; v/v), and 10 ng/ml EGF (Calbiochem, San Diego CA)) [23]. Drops (50 μl) of in vitro maturation culture media were overlayed with embryo tested mineral oil (0.2 μm sterile filtered and stored in the dark). Mouse oocytes and embryos were cultured in an incubator at 37.2°C in 6% CO2 in humidified air.

Methods for in vitro maturation, in vitro fertilization, and embryo culture were modifications of those previously published [24,25]. Briefly, oocytes were matured for 17 hours in K-MAT medium supplemented with either vehicle or PGE2. To prevent zona hardening, 1 mg/ml of dialyzed Fetuin [26,27] and 1% FBS (v/v) were added to the FHM collection medium and the in vitro maturation culture media. Sperm were collected from the caudal epididymis of B6D2 F1 males and capacitated in modified Tyrodes medium for 90-120 min prior to fertilization. Following in vitro maturation, oocytes were transferred into mKSOM^aa ^medium (KSOM medium with 1/2× essential and non-essential amino acids, 5.5 mM glucose and 4 mg/ml BSA and capacitated sperm were added (1 × 10^6^/ml). Eggs were removed from the fertilization dishes after 6 hours of sperm exposure and checked for pronuclei, gently washed to remove loose sperm, and cultured in 50 μl drops of mKSOM^aa ^under oil. After 24 hours of culture, eggs were examined for cleavage to 2 cells. All 2-cell embryos were washed and transferred to fresh culture plates of mKSOM^aa ^to remove the dead sperm. Embryo development was evaluated at 120 hours after sperm addition, and progression to blastocyst formation and zona hatching was recorded.

#### Immunofluorescent detection of EP receptors

Methods for fixation and immunohistochemistry were similar to those previously reported [28]. Briefly, oocytes with minimal cumulus cells were fixed for 10 min at room temperature in FHM medium with 3% paraformaldehyde (w/v) followed by 30 min at 35°C in microtubule stabilization buffer with 2% formalin (w/v). After fixation, eggs and embryos were permeabilized for 5 min in 0.1% Triton-X100 (v/v) in wash solution [28]. Primary antibodies for EP receptor localization were as described for monkey oocytes. Oocytes were incubated with primary antibodies overnight at 4°C followed by secondary antibody for 2 hours at 35°C. Oocytes were then transferred to wash solution containing 10 μg/ml Hoechst 33258 (DNA) with 1:100 phalloidin-Alexa 568 (*f*-actin; Molecular Probes-Invitrogen, Carlsbad, CA) for 1 hour and mounted on glass slides (mounting medium consisted of 1:1 glycerol: PBS supplemented with 5 mg/ml sodium azide and 10 μg/ml Hoechst 33258). All blastocysts were fixed for 30 min in 2% paraformaldehyde (w/v) in PBS (pH 7.4) and labeled for 1 hour with 1:100 Calponin-3 (Santa Cruz Biotech, Santa Cruz CA) to label the inner cell mass, followed by 1 hour with goat anti-mouse Alexa-488 secondary then 30 min with Hoechst 33258 and phalloidin-Alexa 568. Oocytes and embryos were imaged immediately after staining. Serial z-sections (1 μm depth) were obtained using a Zeiss Pascal confocal microscope.

#### RT-PCR

Total RNA was isolated from oocytes using Trizol reagent and was reverse transcribed using random primers [29]. The resulting cDNA was amplified in PCR reactions using primers specific to murine EP1, EP2, EP3 and EP4 and Power Sybr Green Master Mix (Applied Biosystems). GAPDH and 18 S Taqman primers and probe sets (Applied Biosystems) were used to confirm the relative amounts of cDNA within the samples. The primer pairs used are as follows: EP1, forward: 5'-GCAGCACTGGCCCTCTTG-3'; reverse: 5'-TGTGCCATTATCGCCTGTTG-3'; EP2, forward: 5'-GCCTTTCACAATCTTTGCCTACAT- 3', reverse: 5'-GACCGGTGGCCTAAGTATGG-3'; EP3, forward: 5'-GGTCGCCGCTATTGATAATGA -3', reverse: 5'-TCCCATCTGTGTCTTGCATTG -3'; and EP4, forward: 5'-GCTCGTGGTGCGAGTGTT-3', reverse: 5'-CTGGGTTTCTGCTGATGTCTTTC-3'. In all cases, primer pairs were designed to span an intron to prevent amplification of genomic DNA. Additionally, dissociation curves were performed to monitor for potential non-specific amplifications. The standard cycling conditions were 50°C for 2 min, 95°C for 10 min, followed by 40 cycles of 95°C for 15 sec and 60°C for 1 min.

#### Cyclic AMP generation

Denuded oocytes were transferred to oocyte maturation medium supplemented with one of 4 treatments (10^-6^M PGE2, 10^-5^M butaprost, 10^-7^M PGE1 alcohol, or vehicle (0.06% DMSO; v/v)) and cultured for 3 hours. Media + oocytes were then stored at -20°C until processed for determination of cAMP as described for monkey oocytes above.

#### Data analysis

Data were assessed for heterogeneity of variance using Bartlett's test. Cyclic AMP data were log transformed prior to analysis. Cyclic AMP data as well as monkey oocyte maturation data were analyzed by ANOVA, followed by posthoc analysis with Duncan's Multiple Range Test. Comparisons of monkey oocyte maturation between treatment groups, fertilization rates of monkey oocytes, and progression of mouse oocytes to GVBD were assessed by paired t-test. All parametric tests were performed with StatPak v4.12 software (Northwest Analytical, Portland, OR). Mouse oocyte maturation and embryonic development were assessed by Fisher's exact test (Prism 4.01, Graphpad Software Inc., La Jolla, CA). Significance was assumed at p < 0.05. Data are presented at mean ± standard error of the mean (SEM).

## Results

### EP expression in monkey oocytes

**Figure 1:**
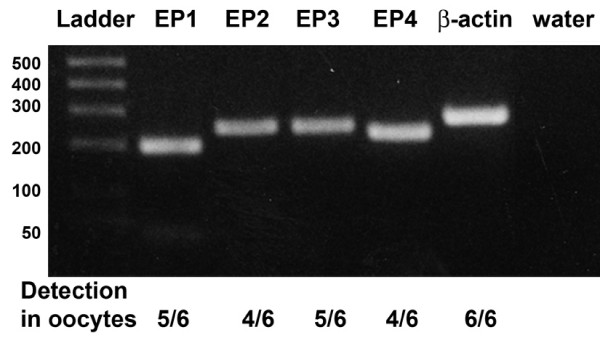
**Detection of EP mRNA in monkey oocytes**. Products of the expected size were obtained by RT-PCR for EP1 (203 base pair (bp)), EP2 (253 bp), EP3 (251 bp), EP4 (228 bp), and β-actin (270 bp). Amplified product was not obtained when water was substituted for reverse transcribed monkey RNA. Position of DNA base pair standards shown on left (bp). Below gel, the number of oocyte preparations with detectable EP receptor mRNA is shown out of the total of 6 preparations tested; β-actin mRNA was detected in all 6 preparations tested.

RNA obtained from monkey oocytes was assayed for each EP receptor by RT-PCR. EP receptor mRNAs were detected in the majority of monkey oocyte preparations (Figure 1). Aromatase mRNA was not detected in any of the 6 oocyte preparations (not shown), indicating that granulosa cell contamination was not responsible for detection of EP mRNAs in monkey oocytes.

Immunofluorescence and confocal microscopy were used to detect individual EP receptor proteins in monkey oocytes and determine the location of each EP receptor within the oocyte.

**Figure 2:**
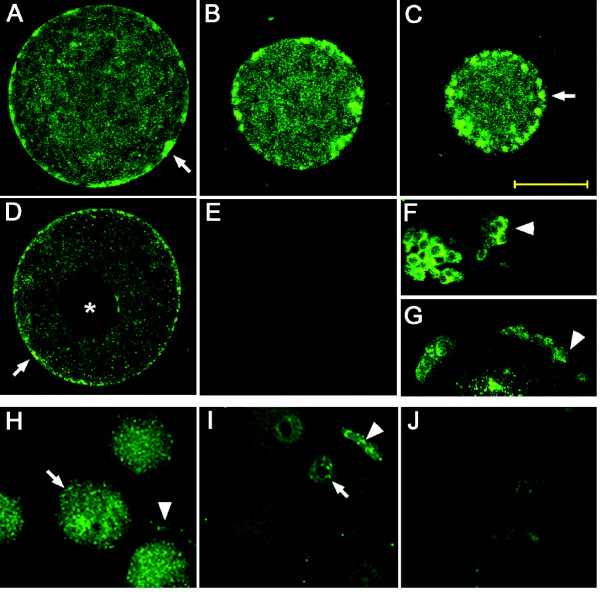
**Immunofluorescent detection of EP receptor proteins (green) in monkey and mouse oocytes**. Panels A-C. EP2 immunofluorescence in representative serial sections through a single monkey oocyte demonstrating concentration of fluorescence at the plasma membrane (arrows), with diffuse central staining. Panel D. EP4 immunofluorescence was present primarily concentrated at the monkey oocyte plasma membrane, with limited central fluorescence; germinal vesicle did not stain as indicated with an asterisk (*). Immunofluorescent detection of EP2 (F) and EP4 (G) in monkey cumulus granulosa cells is indicated by arrowheads. Immunofluorescence was reduced when primary antibody for EP2 (Panel E) or EP4 (not shown) was preabsorbed with the antigenic peptide. Omission of a primary antibody resulted in no staining (not shown). Panels H-J. Immunodetection of EP2 (H) and EP4 (I) in mouse oocytes (arrows) and surrounding cumulus (arrowheads). Immunofluorescence representing detection of EP2 was observed throughout the oocyte cytoplasm, while EP4 immunofluorescence was concentrated near the germinal vesicle (GV). Immunofluorescence was reduced in both oocytes and cumulus when the primary antibody for EP2 (J) or EP4 (not shown) was preabsorbed with the antigenic peptide; omission of the primary antibody yielded very low staining (not shown). For both species, images are representative of a minimum of 2 oocytes from a minimum of 3 different animals. Bar in Panel C = 50 μm and should be used for all Panels.

EP2 receptor protein was detected in each oocyte examined. EP2 immunofluorescence was strong in the region of the plasma membrane, with some immunofluorescence in the central portion of the oocyte (Figure 2A, B, C). Serial sections through an individual oocyte obtained by confocal laser microscopy demonstrated that EP2 immunostaining was present in patches on or near the oocyte surface. Bright cytoplasmic immunofluorescence located near the plasma membrane is consistent with the presence of EP2 in vesicles. Cumulus granulosa cells showed EP2 immunofluorescence (Figure 2F), consistent with previous reports of EP2 expression by cumulus cells [10] and serving as a positive control.

EP4 receptor protein immunofluorescence was observed at the periphery of the oocyte, consistent with the anticipated location of EP4 in the plasma membrane (Figure 2D). Bright cytoplasmic immunofluorescence located in the oocyte cytoplasm near the plasma membrane is consistent with the presence of EP4 in vesicles. Cumulus granulosa cells showed EP4 immunofluorescence (Figure 2G), consistent with reports of EP4 expression by cumulus cells [10] and serving as a positive control.

EP1 receptor protein was not detected in monkey oocytes, with little or no immunofluorescence observed after incubation with EP1 receptor antibody or EP1 receptor antibody preabsorbed with the antigenic peptide (not shown). Specific immunofluorescent detection of EP3 receptor protein in oocytes was also not observed; diffuse fluorescence was observed throughout the oocyte and was only slightly diminished in the center of the oocyte by preabsorption of the primary antibody (not shown).

### Monkey oocyte responses to EP receptor agonists

**Figure 3:**
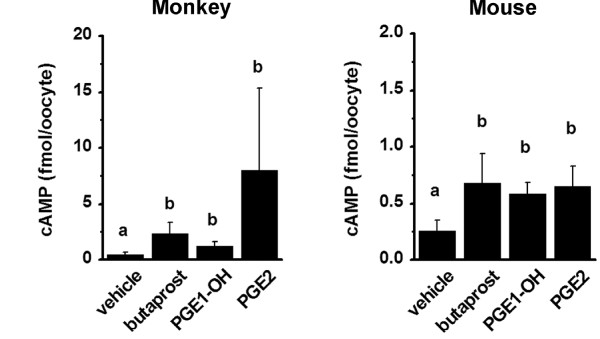
**Cyclic AMP generation in response to EP receptor agonists**. Germinal vesicle (GV) stage oocytes obtained from monkeys (6-10 oocytes/group) and mice (15-20 oocytes/group) were pooled and maintained in culture for 4 hours in the presence of vehicle, the EP2 selective agonist butaprost, the EP4 selective agonist PGE1-OH, or PGE2. Media + oocytes were stored at -20°C until assayed for cAMP by EIA. Data are expressed as fmol cAMP/oocyte. Within each species, data were analyzed by ANOVA and Duncan's Multiple Range Test. Groups with different letters are different, p < 0.05. Data are expressed as mean + SEM, n = 3-10 groups of oocytes/treatment.

To determine if monkey oocytes express functional EP receptors, GV stage oocytes stripped of cumulus granulosa cells were cultured with PGE2, the EP2 receptor agonist butaprost, or the EP4 receptor agonist PGE1 alcohol. All three EP agonists increased cAMP levels in oocyte cultures when compared with cultures receiving vehicle only (Figure 3).

**Figure 4:**
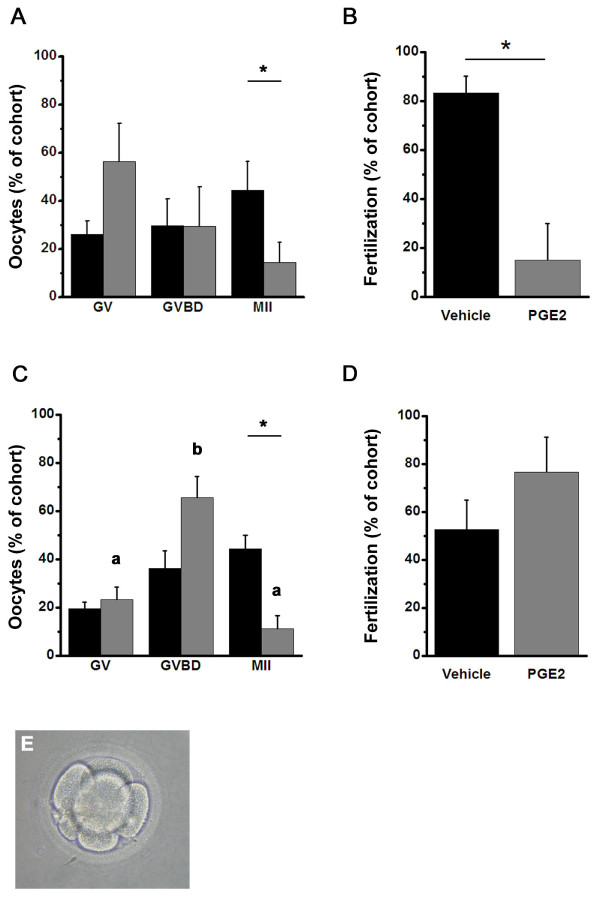
**Monkey oocyte maturation and fertilization in vitro**. Panels A-B. Cumulus-denuded oocytes (6-15 oocytes/animal; n = 4 animals) retrieved at the GV stage were maintained in culture with either vehicle or PGE2. A. Stage of oocyte nuclear maturation was assessed after 30 hours in culture as either GV intact, germinal vesicle break down (GVBD), or meiosis II (MII). B. Fertilization was expressed relative to the total number of oocytes which reached the MII stage by the morning after sperm addition. Panels C-D. Oocytes with intact cumulus (10-12 oocytes/animal; n = 3 animals) were cultured with vehicle or PGE2; cumulus was removed for assessment of nuclear maturation and fertilization. C. Stage of oocyte nuclear maturation was assessed after 30 hours in culture. D. Fertilization was expressed relative to the total number of oocytes which reached the MII stage by the morning after sperm addition. In Panels A-D, black bars represent vehicle-treated oocytes and grey bars represent PGE2-treated oocytes. Data in Panels A and C were analyzed by ANOVA and Duncan's Multiple Range Test. In Panel C, groups with different letters are different. In Panels A and C, vehicle is different from PGE2 treatment as determined by paired t-test and indicated by an asterisk (*). Data in Panels B and D were analyzed by paired t-test, and differences are indicated by an asterisk (*). Data are expressed as a percentage (mean + SEM) of the total number of oocytes in each treatment group for each animal (cohort). For all tests, significance is assumed at p < 0.05. Representative monkey embryo obtained from an oocyte treated in culture with PGE2 (Panel E).

To determine if PGE2 can act directly at monkey oocytes to alter oocyte nuclear maturation and fertilization, oocytes obtained at the GV stage were stripped of cumulus cells and cultured with PGE2 or vehicle (Figure 4A). After 30 hours of culture, 45 ± 12% of vehicle-treated oocytes reached the MII stage while only 15 ± 8% of the PGE2-treated oocytes reached MII (p < 0.05). Because GVBD stage oocytes can continue nuclear maturation in vitro [30], GVBD and MII stage oocytes were transferred to fresh media without vehicle or PGE2 and exposed to sperm in vitro. All oocytes that reached MII by morning were assessed for fertilization. A second polar body, evidence of successful fertilization, was observed in a higher percentage of vehicle-treated oocytes when compared to PGE2-treated oocytes (Figure 4B).

To determine if intact cumulus cells alter oocyte responses to PGE2, additional GV-stage oocytes were cultured with intact cumulus in the presence of either vehicle or PGE2 for 30 hours. 44 ± 6% of cumulus-intact oocytes reached the MII stage during vehicle treatment while only 11 ± 6% of cumulus-intact oocytes reached the MII stage during PGE2 treatment (p < 0.05; Figure 4C). Cumulus cells were then removed from GVBD and MII stage oocytes by gentle pipetting. Oocytes were transferred to fresh media without vehicle or PGE2 and fertilized in vitro. Of the oocytes that reached MII by morning, similar percentages of vehicle- and PGE2-treated oocytes were successfully fertilized (Figure 4D).

### EP expression in mouse oocytes

The data presented above indicate that oocytes from a non-human primate species, the cynomolgus macaque, express EP2 and EP4 receptors coupled to Gαs. Exposure to the endogenous hormone (PGE2) and ligands selective for EP2 and EP4 receptors (butaprost and PGE1-alcohol, respectively) increased cAMP levels, and PGE2 influenced nuclear maturation and fertilization rates of monkey oocytes in vitro.

Similarly, expression and function of EP receptors in mouse oocytes were examined (not shown). EP3 and EP4 mRNAs were detected by RT-PCR in all mouse oocyte preparations examined (n = 3), and EP2 mRNA was detected in 1 of 3 oocyte preparations. In contrast, EP1 mRNA was not detected in any mouse oocyte preparation examined (n = 3).

Interestingly, all mouse oocytes expressed EP2 and EP4 proteins, though EP2 mRNA was only detectable in 1 of 3 oocyte samples. Confocal laser microscopy detected EP2 protein in the cytoplasm of oocytes (Figure 2H). EP4 was also detected in mouse oocytes and was located primarily near the oocyte nucleus (Figure 2I). Cumulus granulosa cells showed EP2 and EP4 immunofluorescence (Figure 2H), confirming previous reports and serving as a positive control [10]. EP1 and EP3 were not observed in mouse oocytes (not shown), similar to results obtained with monkey oocytes. Preabsorption of the primary antibody with the corresponding peptide eliminated immunofluorescence (Figure 2J and not shown), and oocytes incubated without primary antibody were also devoid of immunofluorescence (not shown).

### Mouse oocyte responses to EP receptor agonists

Mouse oocytes expressed functional EP receptors. Mouse oocytes stripped of cumulus granulosa cells were cultured with PGE2, butaprost, or PGE1 alcohol; all EP agonists increased cAMP levels in oocyte cultures when compared with cultures of oocytes treated with vehicle only (Figure 3).

**Figure 5:**
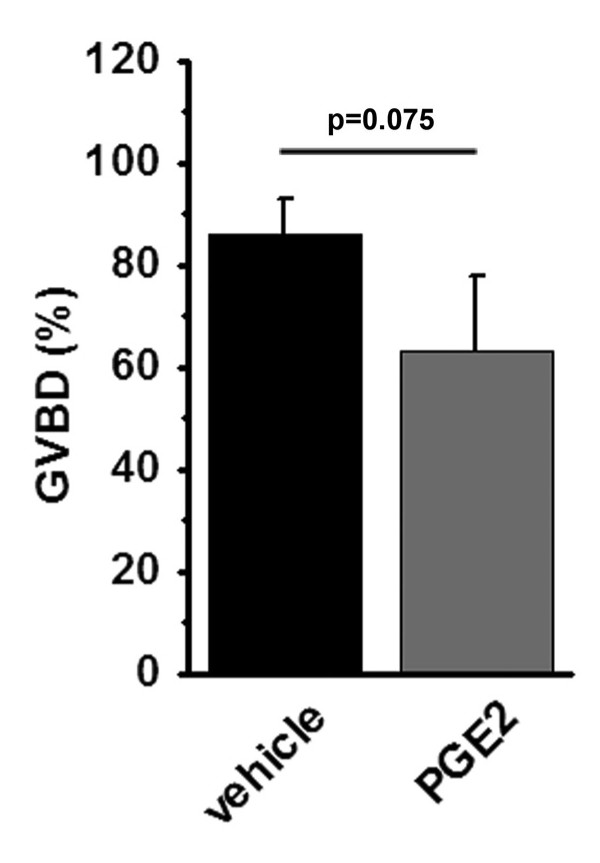
**Mouse oocyte maturation in vitro**. Mouse oocytes retrieved at the GV stage were maintained in culture with vehicle or PGE2 for 2 hours and assessed for progression to GVBD. Data are expressed as mean + SEM, n = 4 animals. Rate of progression to GVBD in the presence of PGE2 tended to be lower than for vehicle-treated oocytes by paired t-test, p = 0.075.

To determine if PGE2 treatment of culture mouse oocytes altered nuclear maturation or fertilization, oocytes obtained at the GV stage were maintained in vitro with vehicle or PGE2. After 2 hours of culture, 86% of vehicle-treated oocytes had completed GVBD; during this same culture period, 63% of PGE2-treated oocytes completed GVBD, with a trend toward decreased nuclear maturation with PGE2 treatment (p = 0.075; Figure 5). PGE2 treatment also resulted in significantly decreased progression to MII after 18 hours in vitro (Table 1). Similar percentages of vehicle- and PGE2-treated oocytes fertilized successfully, formed blastocysts, and hatched in vitro (Table 1).

**Table 1:** Mouse oocyte in vitro maturation and fertilization

	Vehicle (%)	PGE2 (%)
MII at 16 hours	53	69
MII at 18 hours	95	84^a^
Fertilization (2 cells)	71	71
Blastocyst	57	63
Hatched blastocyst	29	37

Each observation represents the percentage of all oocytes in the indicated treatment group. Oocytes were pooled from 2 animals, n = 36-69 oocytes per treatment group. ^a^Percent of cohort which progressed to MII by 18 hours was lower with PGE2 treatment, p < 0.05 by Fisher's exact test.

## Discussion

The present study demonstrates that mammalian oocytes express functional receptors for PGE2. Multiple EP receptor mRNAs were detected in monkey and mouse oocytes, but only EP2 and EP4 receptor proteins were consistently detected by immunofluorescence. These discrepancies are reminiscent of our studies of EP expression in monkey mural granulosa cells before administration of the ovulatory gonadotropin stimulus, when EP1 and EP4 mRNA were detected, but these EP proteins were not detected by immunofluorescence [8]. EP3 and EP4 mRNA were detected in all mouse oocytes examined, while only 1 of 3 oocyte preparations had detectable EP2 mRNA. A previous report suggested that EP2 and EP4 receptors were present in the cumulus, but not oocyte, of the mouse cumulus-oocyte complex [10]. Our detection of EP receptor proteins within the oocyte, with inconsistent detection of EP mRNAs, might suggest that cumulus cells are the source of these EP receptor proteins. However, use of confocal microscopy in the present study permitted localization of EP receptors specifically to oocytes of mice and monkeys, suggesting that mammalian oocytes express receptors capable of direct response to PGE2.

Previous studies by this laboratory and others focused on granulosa cells as the primary target for PGE2 action within the periovulatory follicle. Mural cells are well established as expressing multiple EP receptors. Granulosa cells from monkey periovulatory follicles expressed mRNA for all 4 EP receptors, with EP1, EP2, and EP3 receptor proteins detected by immunofluorescence [8]. Granulosa cells from human follicular aspirates were reported to express mRNA for EP2 and EP4, while EP1 and EP3 mRNA were not detected [31]. The present study confirmed that monkey and mouse cumulus granulosa cells expressed EP2 and EP4 receptor proteins. Previously, Segi and colleagues used in situ hybridization to successfully localize mRNA for EP2 and EP4 (but not EP1 and EP3) to cumulus granulosa cells within mouse periovulatory follicles [9]. More recently, EP2, EP3 and EP4 mRNA and proteins were localized to mouse cumulus cells [10]. While the present study shows that EP2 and EP4 receptor proteins are expressed by mouse cumulus, further studies will be needed to confirm expression of EP3 receptor proteins. It is possible that cumulus cell expression of EP receptors changes in response to the midcycle LH surge, which may explain discrepancies between these studies. Recently, theca cells have also been suggested as a target for PGE2 action, with detection of mRNA for EP2, EP3, and EP4 in bovine theca [32] and EP2 mRNA in mouse theca cells [9]. Taken together, these findings support the concept that multiple cell types within the periovulatory follicle, including cumulus granulosa cells, mural granulosa cells, theca cells, and now oocytes, are potential targets for PGE2 action.

EP receptors utilize G proteins to mediate the effect of PGE2 binding to these transmembrane receptors [7]. EP2 and EP4 are reported to couple exclusively with Gαs to activate adenylyl cyclase and increase intracellular cAMP. In both monkey and mouse oocytes, stimulation of EP2 and EP4 receptors increased cAMP as anticipated. The distribution of EP2 receptors at the surface of monkey oocytes was patchy, while EP4 receptors were distributed more uniformly around the surface of monkey oocytes. Microdomains have been reported in the vicinity of plasma membranes, wherein a receptor associates with specific G proteins, membrane-associated enzymes, and other components of the intracellular signaling apparatus [33]. Microdomains present the possibility that EP2 and EP4 receptors may preferentially associate with different isoforms of adenylyl cyclase or cAMP phosphodiesterases in oocyte plasma membranes. In mouse oocytes, EP2 was localized throughout the cytoplasm while EP4 was primarily perinuclear in location. Although prostaglandin receptors are often located in plasma membranes, presence of prostaglandin receptors, including EP4, in perinuclear membranes has been reported [34]. Since EP2 and EP4 receptors were differentially distributed within monkey and mouse oocytes, cAMP generated via each receptor may have a unique function. Furthermore, species differences in EP2 and EP4 distribution suggests that PGE2 may regulate different functions in oocytes obtained from different mammalian species.

Stimulation of EP2 and EP4 receptors increased oocyte cAMP levels. Levels of cAMP measured in this study were similar to previous reports of basal, PGE1-, and PGE2-stimulated cAMP in mouse and bovine oocytes [35,36]. Many pathways have been suggested to elevate oocyte cAMP levels, including ligand-independent receptors coupled to Gαs and adenylyl cyclase [37,38], suppression of cAMP phosphodiesterase activity to limit cAMP breakdown [39], and transfer from granulosa cells [40]. In vivo, cumulus cells surrounding oocytes also express EP2 and EP4 receptors, and cAMP generated within cumulus cells can move to the adjacent oocyte through junctional complexes connecting these cells. The actions of PGE2 on individual cell types within the oocyte-cumulus complex are difficult to discern in vivo. However, these studies do support the hypothesis that oocytes can respond directly to PGE2 via stimulation of EP2 and EP4 receptors to contribute to oocyte cAMP concentrations.

Levels of cAMP within the oocyte are critical in the control of resumption of meiosis. The present study shows that PGE2 treatment delayed progression of meiosis in mouse oocytes as well as in monkey oocytes without and with surrounding cumulus. This observation is consistent with the concept that high cytoplasmic cAMP within the oocyte prevents resumption of meiosis; low cAMP permits meiosis to continue [41]. Several agents which increased adenylyl cyclase-generated cAMP levels also delayed resumption of meiosis in bovine oocytes without and with surrounding cumulus [36]. When mouse oocytes were treated in vitro with cholera toxin or PGE1, trends towards increased cAMP and delayed resumption of meiosis have been reported [35]. These findings, taken together with data from the present study, suggest that elevated PGE2 within the follicle may delay resumption of meiosis in vivo, with more pronounced effects in species with longer intervals between the midcycle LH surge and ovulation. This concept is consistent with previous studies showing delayed induction of COX-2 expression and prostaglandin synthesis in follicles of species with long periovulatory intervals [1,42-44].

Fertilization and subsequent embryonic development of denuded mouse oocytes was not altered by PGE2 treatment. This finding is consistent with previous studies showing that in vitro fertilization rates for oocytes stripped of surrounding cumulus cells were similar between wild type mice and mice lacking EP2 receptor expression [5]. Overall, elevated follicular PGE2 may delay nuclear maturation somewhat, but PGE2 exposure does not alter fertilization of mouse oocytes.

In contrast, PGE2 treatment of monkey oocytes without surrounding cumulus had a negative impact on the fertilization rate. Interestingly, PGE2 treatment of cumulus-intact oocytes did not alter subsequent fertilization. PGE2 treatment reduced oocyte nuclear maturation in the absence and presence of surrounding cumulus, suggesting that progression of meiosis is modulated by PGE2 action directly at oocytes. In the context of the periovulatory follicle, PGE2 action at EP receptors on cumulus cells and elsewhere in the follicle may directly or indirectly regulate critical maturational changes within the oocyte which promote successful fertilization.

## Conclusions

These data demonstrate that mammalian oocytes express functional PGE2 receptors. Since PGE2 levels in the follicle increase in response to the midcycle LH surge, PGE2 likely reaches EP receptors on both the oocyte and surrounding cumulus cells prior to ovulation. The ability of PGE2 to delay oocyte maturation was more pronounced in non-human primate oocytes than in murine oocytes, suggesting that PGE2 may play a physiologic role to slow oocyte maturation in species with longer intervals between the LH surge and ovulation. PGE2 may be one of several local signaling molecules within the follicle which coordinate oocyte maturation with the time of follicle rupture, ensuring release of an optimally-mature oocyte at ovulation.

## Competing interests

The authors declare that they have no competing interests.

## Authors' contributions

DMD conceived of the monkey oocyte/embryo studies, conducted monkey oocyte/embryo studies, performed cyclic AMP assays, and drafted the manuscript. LKMG conducted mouse oocyte/embryo studies. CAVV provided monkey sperm and advised on optimation of monkey IVF and oocyte/embryo culture. LKC conceived of the mouse oocyte/embryo studies, performed RNA amplification/RT-PCR for mouse and monkey oocytes, and helped to draft the manuscript. All authors read and approved the final manuscript.
